# Live Birth in a 50-Year-Old Breast Cancer Survivor Using a Cryopreserved Embryo: A Case Report

**DOI:** 10.7759/cureus.93319

**Published:** 2025-09-26

**Authors:** Sagiri Taguchi

**Affiliations:** 1 In Vitro Fertilization Center, Oak Clinic, Osaka, JPN

**Keywords:** assisted reproductive technology (art) cycles, breast cancer survivor, cryopreserved embryo, embryo banking, infertility, live birth, oncofertility

## Abstract

This report describes the rare case of a woman who, after starting infertility treatment at age 44 and subsequently being diagnosed with breast cancer, successfully achieved a live birth at age 50. The patient underwent numerous assisted reproductive technology cycles, ultimately conceiving with a cryopreserved embryo that was created when she was 45 and stored for five years. To our knowledge, this is the first reported case of a live birth in a breast cancer survivor at the age of 50, achieved through the use of her own cryopreserved embryo. This case represents a remarkable success achieved despite the combined challenges of advanced maternal age and a cancer diagnosis, highlighting the importance of persistent embryo banking and oncofertility.

## Introduction

Female fertility declines significantly in the late 30s, and achieving a live birth using autologous oocytes beyond the age of 45 is exceedingly difficult [1]. This biological limitation is primarily due to a decrease in the number and quality of oocytes, particularly an increase in chromosomal aneuploidy [2]. Consequently, even with assisted reproductive technology (ART), live birth rates for women in their late 40s are in the low single digits, and success at age 50 is so rare as to be the subject of case reports [3]. An additional challenge is faced by women diagnosed with cancer at a young age. Cancer treatments can inflict irreversible damage on ovarian function, leading to diminished fertility post-treatment [4]. In response, oncofertility, which involves cryopreserving oocytes or embryos before initiating cancer therapy, has become a standard of care to preserve future reproductive options [5,6]. For patients with hormone receptor-positive breast cancer, the need for long-term adjuvant endocrine therapy can further delay pregnancy attempts, compounding the effect of aging [7].

This case report describes the exceptional case of a patient who overcame these multiple barriers. To my knowledge, this is the first report of a breast cancer survivor achieving a live birth at age 50. This success was accomplished through the use of a long-term cryopreserved embryo, a testament to the patient’s resilience and the efficacy of modern ART strategies, offering valuable insights for the clinical community.

## Case presentation

The patient began infertility treatment in 2009 at the age of 44. Her partner was nine years older, and cryopreserved sperm was used for all intracytoplasmic sperm injection procedures due to availability issues. Over the course of six years, she underwent a total of 21 oocyte retrieval cycles and seven frozen-thawed embryo transfer (FET) cycles. A persistent strategy of oocyte and embryo banking was employed to accumulate a sufficient number of embryos. Her treatment history is summarized in Table 1.

**Table 1:** Summary of treatment history (2009–2015) FET: frozen-thawed embryo transfer

Period	Patient age	Treatment	Details and outcome
2009	44	Fresh embryo transfer and embryo banking	First oocyte retrieval and fresh embryo transfer resulted in a chemical pregnancy (hCG 7.46). Subsequently, 12 embryos were cryopreserved over five cycles.
2010	45	Oocyte and embryo banking	Due to a period of partner unavailability, 12 oocytes were cryopreserved over 10 cycles. Later, an additional oocyte retrieval, combined with the thawing of cryopreserved oocytes, resulted in four cryopreserved embryos. The embryo that resulted in the live birth was created this year.
2011–2012	46–47	FET	Two FET cycles were performed (three embryos transferred each time); both failed to result in pregnancy.
Late 2012	47	Embryo banking and breast cancer diagnosis	Three additional embryos were cryopreserved over four retrieval cycles. In September, she was diagnosed with left-sided breast cancer and underwent surgery in October.
2013–2014	48–49	FET	Post-cancer treatment, she resumed FET cycles. Four attempts (transferring three, two, two, and two embryos, respectively) were unsuccessful.
2015	49–50	FET and live birth	In February, two cryopreserved embryos were transferred, resulting in a singleton pregnancy. In October, at age 50, she delivered a healthy boy via Cesarean section.

**Figure 1:**
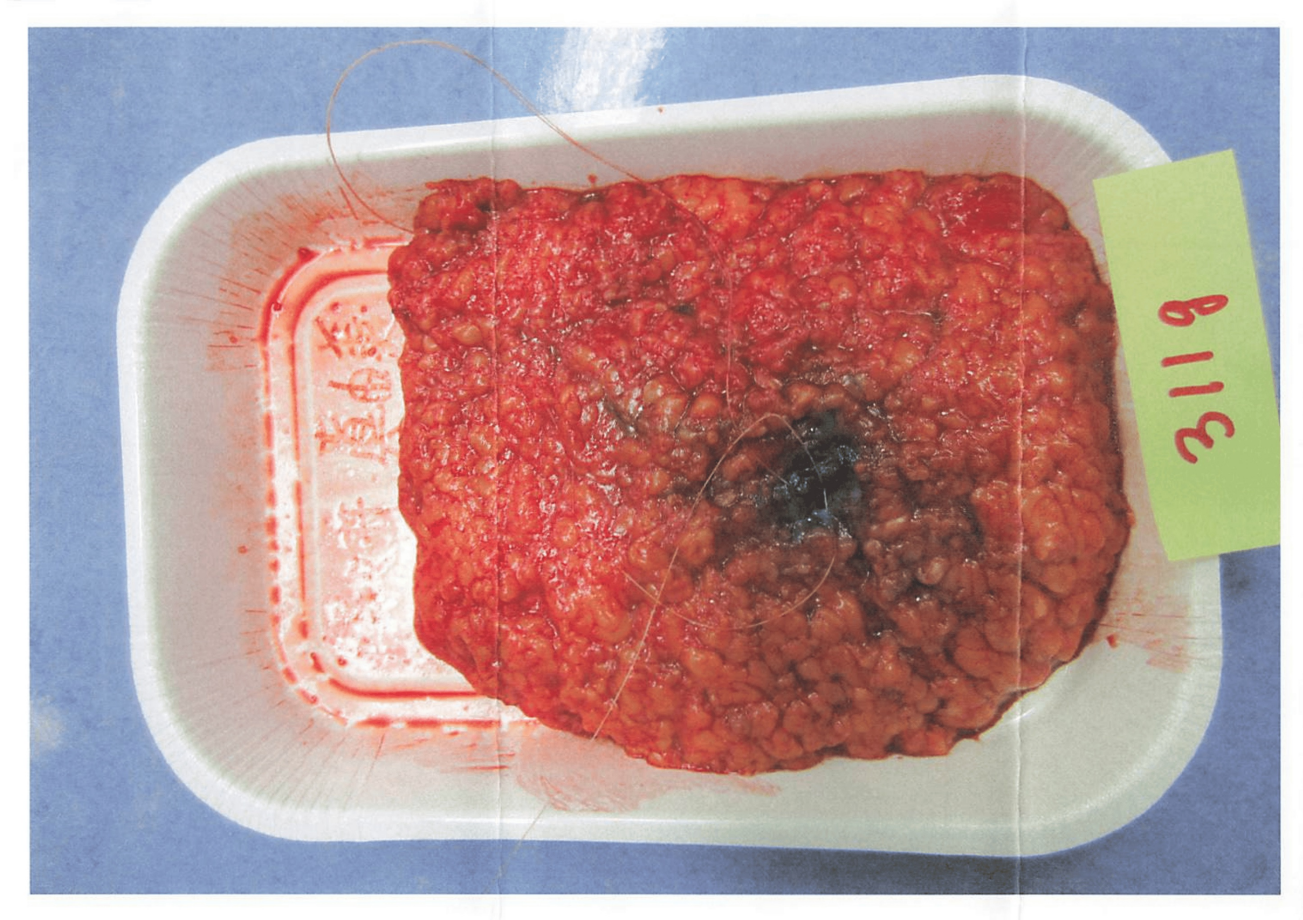
Resected breast tissue specimen following breast cancer surgery

In September 2012, at age 47, she was diagnosed with ductal carcinoma in situ (DCIS) of the left breast. She underwent a left subcutaneous mastectomy with sentinel lymph node biopsy and immediate reconstruction on October 31, 2012. Pathology showed a 12 mm lesion, ER(+), PgR(+), HER2(-), nuclear grade 2, comedo type, with negative margins and no lymph node involvement. The histopathological findings are shown in Table 2. Furthermore, an image after breast cancer surgery is shown in Figure 1.

**Table 2:** Histopathological findings of the breast cancer DCIS: ductal carcinoma in situ

Feature	Finding
Diagnosis	DCIS
Major diameter	12 mm
Histological grade	Nuclear grade 2, comedo type
Hormone receptors	ER (+), PgR (+)
HER2 status	Negative (-)
Surgical margins	Negative (-)
Lymph node status	No metastasis (pN0, 0/1)

Importantly, no chemotherapy, radiotherapy, or endocrine therapy (e.g., tamoxifen or aromatase inhibitors) was administered after surgery. This allowed her to resume fertility treatment without additional delays that such therapies often cause.

After cancer treatment, she resumed ART cycles. Following six unsuccessful FET attempts, two embryos cryopreserved in 2010 (at age 45) were thawed and transferred in a hormone replacement cycle in February 2015. Serum hCG was 446.6 mIU/mL, confirming pregnancy. Details of the successful cycle are presented in Table 3.

**Table 3:** Details of the successful FET cycle (February 2015) HRT: hormone replacement therapy, FET: frozen-thawed embryo transfer

Parameter	Details
Endometrial preparation	HRT cycle
Medications used	Estrana®, Lutinus® vaginal suppositories
Transferred embryos	Two cleavage-stage embryos cryopreserved in 2010 (patient age at cryopreservation: 45)
Pregnancy test (serum hCG)	446.6 mIU/mL
Pregnancy course	Singleton, uneventful

The pregnancy course was uneventful despite the patient’s advanced maternal age and primiparity. She was closely monitored throughout for complications such as gestational hypertension and diabetes, but none developed. At 38 weeks and five days, she delivered a healthy male infant via elective cesarean section. Perinatal outcomes are summarized in Table 4.

**Table 4:** Perinatal information SD: standard deviation

Parameter	Details
Maternal age at delivery	50 years
Gestational age at delivery	38 weeks, 5 days
Mode of delivery	Elective cesarean section
Infant sex	Male
Birth weight	3090 g (+0.53 SD)
Birth length	50.5 cm (+1.03 SD)
Apgar scores	8 (1 min)/9 (5 min)
Notable findings	No external malformations

## Discussion

This case highlights a remarkable achievement in reproductive medicine. First, the live birth at age 50 using an autologous cryopreserved embryo is exceedingly rare, especially in a breast cancer survivor. Although isolated reports exist of live births in women aged 50 without cancer [3], this case represents the first such report in a patient with a history of breast cancer.

Second, this outcome underscores the importance of oncofertility. The strategy of cryopreserving embryos before cancer treatment enabled this patient to pursue pregnancy years later, after completion of oncologic management [5,6,8]. This is particularly relevant for patients with hormone receptor-positive breast cancer, who may otherwise face years of endocrine therapy during which natural fertility declines [7,9]. In contrast, women who do not undergo fertility preservation before treatment typically have almost no chance of achieving biological motherhood at such an advanced age, leaving donor oocytes or adoption as the only feasible options.

Third, this case validates the practice of embryo banking in poor-prognosis patients. Starting with diminished ovarian reserve at age 44, the patient persisted with repeated retrievals, eventually producing a viable embryo. While this case demonstrates that persistence and fertility preservation can yield success, it is critical to emphasize that the overall success rates for women in their late 40s and 50s are extremely low, even with ART. Therefore, this case should be seen as an extraordinary exception rather than a reproducible outcome.

Finally, the patient’s capacity to generate a viable embryo at age 45, coupled with her cancer diagnosis at age 47, raises the possibility of shared genetic predispositions. One possible explanation could be variants in the SAMHD1 gene, which have been implicated in both delayed ovarian aging and increased cancer risk [10]. No genetic testing was performed on this patient, and this remains a theoretical consideration warranting further investigation.

## Conclusions

This is the first reported case of a breast cancer survivor achieving a live birth at age 50 using her own cryopreserved embryo. This success reflects the combined power of persistent embryo banking, fertility preservation, and modern ART. Clinicians should counsel patients early about embryo or oocyte banking if cancer therapy is planned, as this case demonstrates the profound long-term value of such strategies. Additionally, this case represents a rare and exceptional success, and outcomes at advanced maternal age remain generally poor.
